# Hyperexcitability in Spinal WDR Neurons following Experimental Disc Herniation Is Associated with Upregulation of Fractalkine and Its Receptor in Nucleus Pulposus and the Dorsal Root Ganglion

**DOI:** 10.1155/2016/6519408

**Published:** 2016-12-26

**Authors:** Daniel Pitz Jacobsen, Aurora Moen, Fred Haugen, Johannes Gjerstad

**Affiliations:** ^1^National Institute of Occupational Health, Oslo, Norway; ^2^Department of Biosciences, University of Oslo, Norway

## Abstract

*Introduction*. Lumbar radicular pain following intervertebral disc herniation may be associated with a local inflammatory response induced by nucleus pulposus (NP) cells.* Methods*. In anaesthetized Lewis rats, extracellular single unit recordings of wide dynamic range (WDR) neurons in the dorsal horn and qPCR were used to explore the effect of NP application onto the dorsal nerve roots (L3–L5).* Results*. A clear increase in C-fiber response was observed following NP conditioning. In the NP tissue, the expression of interleukin-1*β* (IL-1*β*), colony stimulating factor 1 (Csf1), fractalkine (CX3CL1), and the fractalkine receptor CX3CR1 was increased. Minocycline, an inhibitor of microglial activation, inhibited the increase in neuronal activity and attenuated the increase in IL-1*β*, Csf1, CX3L1, and CX3CR1 expression in NP tissue. In addition, the results demonstrated an increase in the expression of TNF, CX3CL1, and CX3CR1 in the dorsal root ganglions (DRGs).* Conclusion*. Hyperexcitability in the pain pathways and the local inflammation after disc herniation may involve upregulation of CX3CL1 signaling in both the NP and the DRG.

## 1. Introduction

Earlier studies show that leakage of nucleus pulposus (NP) out of the disc after intervertebral disc herniation is associated with a local release of many substances including interleukin-1*β* (IL-1*β*), tumor necrosis factor (TNF), and colony stimulating factor 1 (Csf1) [1–4]. Such cytokines released after intervertebral disc herniation may induce increased neuronal excitability in the primary afferent nerve fibers and reduced axonal speed of conduction and may also promote nerve injury [1, 5, 6]. Thus, intervertebral disc herniation has adverse effects on the sensory pathways.

Peripheral inflammation in animals may be associated with activation of satellite glial cells in the dorsal root ganglion (DRG) [7]. Nerve injury and release of inflammatory substances may also activate microglia in the spinal cord [8–10]. For instance, previous data show that soluble fractalkine (CX3CL1) activates glia and leukocytes through its receptor CX3CR1 [11, 12]. Furthermore, spinal upregulation of CX3CL1 may be associated with mechanical allodynia and thermal hyperalgesia [13]. Thus, one important inflammatory molecule possibly influencing the sensory pathways may be CX3CL1.

Evidence exists that the local inflammation associated with disc herniation as well as microglial activation is dependent on phosphorylation of MAPK p38 [14, 15]. Thus, drugs targeting this kinase may attenuate inflammatory pain [10, 16, 17]. Here, in an animal model mimicking clinical intervertebral disc herniation, we investigate the effect of NP on neuronal activity in the spinal cord and examine the local gene expression. We hypothesize that the interaction between NP cells and neuronal tissue may induce a local upregulation of CX3CL1 signaling and increased nociceptive activity in the pain pathways.

## 2. Materials and Methods

### 2.1. Animals and Surgery

A total of 87 adult inbred female Lewis rats (170–215 g) were used in the present study. Female rats were preferred because allergens are more potent in males. Before use, all rats were initially sedated with isoflurane gas (Baxter International Inc., USA) followed by intraperitoneal administration of 250 mg/mL~2.1 g/kg body weight urethane (Sigma-Aldrich Co., Germany). Absence of hind paw withdrawal and ear wriggling to pinch indicated adequate surgical anesthesia. The rat's core temperature was maintained at 36-37°C by a feedback heating pad.

To expose the sciatic nerve, a 1-2 cm incision was made above the pelvic girdle and a bipolar silver hook electrode was placed proximal to the main branches of the sciatic nerve for electrical stimulation. As previously described [1], a laminectomy, with a 1 mm lateral expansion on the left side for the application of NP, was performed at vertebrae Th13-L1, corresponding to the spinal cord segments L3-S1. The vertebral column was rigidly fixed by clamps rostral and caudal to the exposed spinal cord segments. The meninges, that is, dura mater and arachnoidea, were punctured by a cannula and carefully removed by two tweezers. The animals were euthanized by an overdose of pentobarbital.

### 2.2. In Vivo Electrophysiology

A parylene-coated tungsten microelectrode with impedance 2–4 MΩ (Frederick Haer & Co., USA) was lowered vertically into the left dorsal horn of the spinal cord by an electrically controlled micromanipulator (Märzhäuser Wetzlar GmbH & Co. KG, Germany). The spinal cord segments L3-S1 were identified by the neuronal response to left hind paw finger tapping and pinching. Extracellular single cell recordings of wide dynamic range (WDR) neurons were performed at depths of 50–700 *μ*m from the surface of the spinal cord. Only one cell was studied in each animal. The recorded signals from the headstage were amplified (×5000) with an AC preamplifier, band-pass filtered with half amplitude cut of 500–1250 Hz (NeuroLog by Digitimer Ltd, UK), digitalized with interface CED Micro1401, and displayed on a computer screen by the software CED Spike 2.2 (Cambridge Electronic Design, UK). The sampling frequency was 20 kHz.

The software Spike 2.2 was also used to control the electrical stimuli frequency given to the sciatic nerve by the hook electrode, while a pulse buffer connected to a stimulus isolator unit (NeuroLog System, Digitimer Ltd, UK) controlled the stimuli intensities. Single cell recordings were ensured by examining spike shape and amplitude. Spikes 50–300 ms after stimulus were defined as C-fiber responses. The C-fiber threshold was defined at the beginning of each experiment as the lowest stimulus intensity capable of eliciting a single visible spike in the defined C-fiber timeframe. Every fourth minute a single test stimuli (2 ms rectangular pulse, 1.5x C-fiber threshold) was applied to the sciatic nerve. Six stable recordings varying less than 20% served as a baseline for the subsequent experiments. Only cells with baseline responses of 5–20 spikes were included in the study.

To study the effect of NP conditioning on neuronal activity, NP was harvested from 3 to 4 caudal intervertebral discs of a genetically identical donor rat and applied 0.5–2 mm caudally to the recording electrode, covering the incoming left dorsal nerve roots. Minocycline (MC, M9511, Sigma-Aldrich Co., Germany) was chosen to target MAPKp38 in the glial cells. MC was dissolved in 0.9% NaCl, diluted to a concentration of 5 *μ*g/*μ*L, and topically administered onto the nerve roots (25 *μ*L before and 25 *μ*L after NP application). Four series of electrophysiological experiments were performed: (I) NP conditioning (*n* = 7), (II) NP conditioning together with MC (*n* = 7), (III) MC (*n* = 5), and (IV) NaCl control (*n* = 5). The C-fiber response was followed for 180 minutes after conditioning. In addition, 14 animals were used as donor rats.

### 2.3. Parallel NP Gene Expression Experiments

In order to investigate changes in the expression of our candidate genes in NP tissue, four series of experiments were performed and defined as (I) NP, (II) NP+MC, (III) NP^fat^, and (IV) NP^native^. The tissue in the NP group (*n* = 7) had been in contact with the dorsal nerve roots for 180 minutes, as had the tissue in the NP+MC group (*n* = 7) together with minocycline. Tissue samples for these two groups were harvested directly following the electrophysiological experiments described above. The NP^fat^ group (*n* = 9), which was applied onto neck fat tissue for 180 minutes, served as a control group to investigate whether any possible gene expression changes in the NP group were caused by the tissue-specific properties of the nerve roots or surrounding tissues. NP^native^ tissue (*n* = 10) was frozen directly after isolation from the caudal intervertebral discs of the donor rats and served as control. In addition, 9 extra animals were used for the fat controls.

### 2.4. Ipsi- and Contralateral Dorsal Root Ganglion Gene Expression

We also wanted to investigate the gene expression changes in the DRG following experimental disc herniation. Thus, lumbar DRG L3–L5, giving input to TH13 and L1 through their dorsal nerve roots, were dissected out and frozen on liquid nitrogen. The DRG isolation protocol has been described elsewhere [18]. Two series of DRG experiments were performed: NP (*n* = 7) and native (*n* = 7). In both groups, a laminectomy exposing the dorsal nerve roots was performed three hours before isolation of DRG. In the NP group, NP tissue from the caudal intervertebral discs of a donor rat was applied onto the left dorsal nerve roots immediately after the laminectomy. In total, 7 donor rats were used for DRG gene expression experiments. Both the left and the right dorsal root ganglia were isolated. Left L3, L4, and L5 and right L3, L4, and L5 were pooled separately before gene expression analyses.

### 2.5. qPCR

As previously described [19], total RNA was extracted from frozen (−80°C) NP and DRG tissue by the TRIzol reagent (Life technologies, Inc., Rockville, Maryland, USA), chloroform (Sigma-Aldrich, St. Louis, MO, USA), and isopropanol (Merck, Darmstadt, Germany). RNA was reversibly transcribed by aid of the first-strand cDNA Synthesis Kit for reverse-transcriptase polymerase chain reaction (RT-PCR) (AMV) (Roche Diagnostic, Mannheim, Germany). The qPCR analysis was then performed in two parallels on a StepOnePlus qPCR machine (Applied Biosciences, USA). Primers were designed using Primer Express 2.0 (Applied Biosystems, California, USA) and checked for specificity by performing a BLAST search. Effort was made to design primers without nonspecific binding (the melting curves indicated no biproducts). For more details about the primers (Risskov, Denmark), see Table 1. Target genes were normalized to *β*-actin (internal reference).

### 2.6. Statistics

The spinal nociceptive activity, that is, the C-fiber response, was presented as percent of baseline. The 6 baseline recordings were converted to 2 baseline values (each an average of 3 consecutive responses), and the 45 postbaseline recordings were converted to 9 values (each an average of 5 consecutive responses). To examine the effect of the four types of intervention, NP, NP+MC, MC, and control, on spinal nociceptive response, an analysis of variance, repeated-measure design (rmANOVA), was performed. Because the assumption of sphericity was not met, the degrees of freedom were corrected using a Greenhouse–Geisser correction. The average C-fiber responses from 60 to 180 minutes after conditioning were compared using one-way ANOVA and Tukey's honestly significant difference (HSD) post hoc comparisons. Regarding the gene expression, fold change values for each sample were defined by the expression of the target gene normalized to the expression of the reference gene *β*-actin and the level of the corresponding native tissue. Group means of NP expression values were compared using one-way ANOVA and Tukey's HSD post hoc comparisons, while DRG expressions were compared with an unpaired Student's* t*-test. Statistical analyses were performed by use of SPSS 21 (IBM SPSS inc., USA). A *P* value < 0.05 was set as the level of statistical significance. Data are given as representative examples and means ± SEM.

## 3. Results

**Figure 1:**
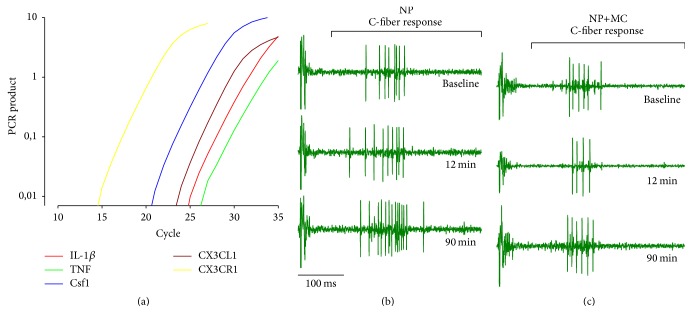
(a) Examples of qPCR amplification plots demonstrating the presence IL-1*β* (red), TNF (green), Csf1 (blue), CX3CL1 (brown), and CX3CR1 (yellow) in native NP tissue. (b) Examples of single cell recordings at baseline, 12 minutes and 90 minutes after nucleus pulposus conditioning alone. (c) Examples of single cell recordings at baseline, 12 minutes and 90 minutes after nucleus pulposus conditioning together with minocycline.

**Figure 2:**
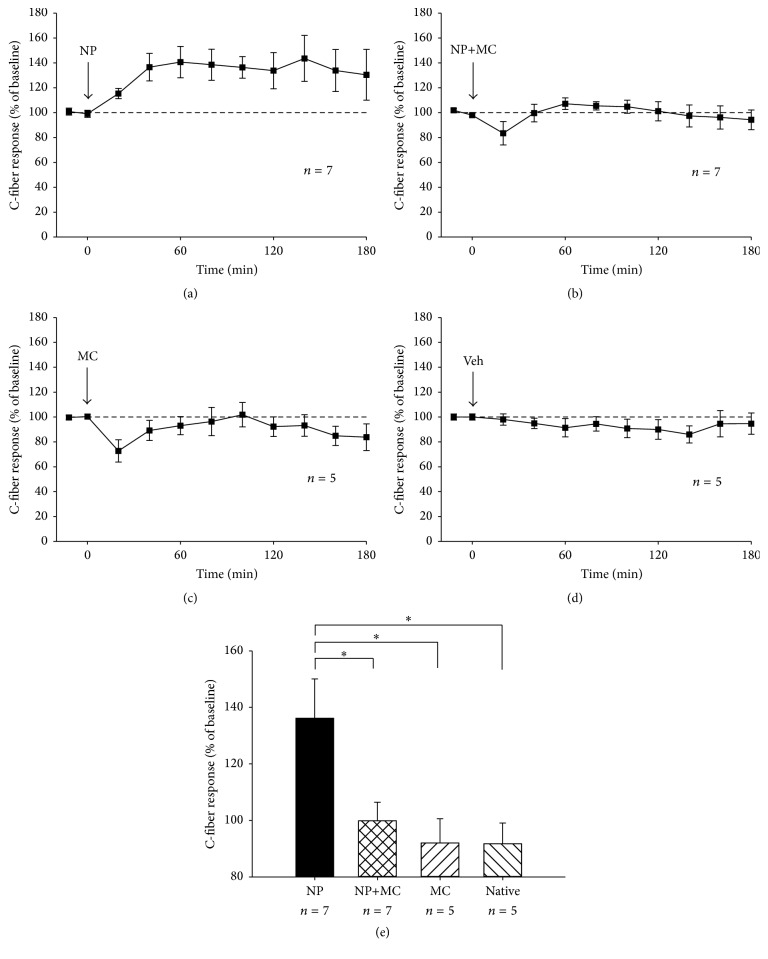
C-fiber response in percent of baseline after application of (a) NP (NP), (b) NP and minocycline (NP+MC), (c) minocycline (MC), and (d) Vehicle (Veh). (e) The mean value 60 to 180 minutes after baseline in the four groups. *P* = 0.018, rmANOVA, four groups; NP, NP+MC, MC, Veh. ^*∗*^*P* < 0.05, one-way ANOVA, Tukey's post hoc test.

We here report that IL-1*β* (red), TNF (green), Csf1 (blue), but also CX3CL1 (brown), and CX3CR1 (yellow) were expressed in the native NP tissue (Figure 1(a)). Moreover, NP applied onto the dorsal nerve roots induced a rapid increase in the C-fiber response (Figure 1(b)), but this was in most cases blocked by minocycline (Figure 1(c)). The observed increase in C-fiber response was evident already 10–20 minutes after NP administration (Figure 2(a)), whereas no clear increase in the C-fiber response was seen following application of NP together with minocycline (Figure 2(b)). Application of minocycline alone caused a short lasting decrease in C-fiber response (Figure 2(c)). No clear changes in the C-fiber response were observed in the vehicle control experiments (Figure 2(d)).

The average C-fiber response 60–180 minutes after NP conditioning was 136.1%  ± 13.9 of baseline, but after NP conditioning together with minocycline only 99.9%  ± 6.5 of baseline. Moreover, the average C-fiber response after minocycline alone was 92.0%  ± 8.6 of baseline, and in the corresponding vehicle control 91.7%  ± 7.4 of baseline (Figure 2(e)). The C-fiber response after conditioning with NP alone was significantly higher than the C-fiber response after conditioning with NP plus minocycline, minocycline alone or in the corresponding vehicle control.

Moreover we observed a clear increase in the expression of IL1*β* in NP samples, but no change in the expression of TNF, and a minor increase in the expression of macrophage colony stimulating factor Csf1 180 minutes after application onto the nerve roots (fold expression: IL-1*β*; 13.47 ± 3.03, TNF 2.89 ± 2.08, Csf1; 3.13 ± 1.21). Notably, a significant increase in the gene expression of CX3CL1 and its cognate receptor CX3CR1 was also observed in the NP tissue (fold expression: CX3CL1; 7.32 ± 1.89, CX3CR1; 7.32 ± 1.89).

**Figure 3:**
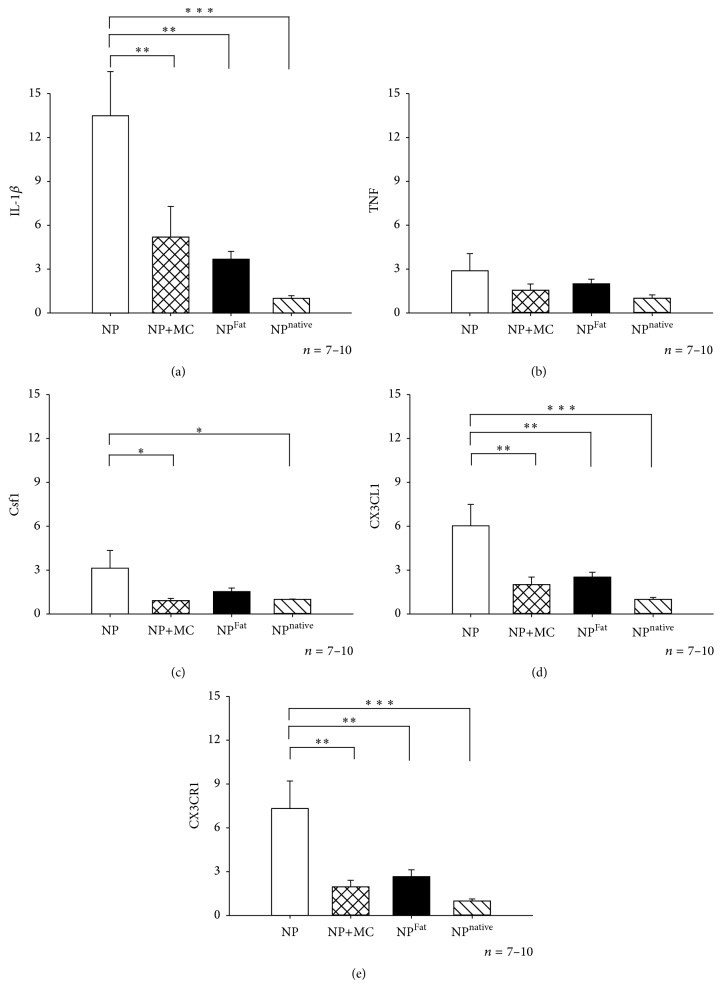
Fold expression of (a) IL-1*β*, (b) TNF, (c) Csf1, (d) CX3CL1, and (e) CX3CR1 in NP in the three groups: NP in contact with dorsal nerve roots for 180 minutes (NP), NP in contact with dorsal nerve roots for 180 minutes together with minocycline (NP+MC), NP in contact with neck fat tissue for 180 minutes (NP^Fat^), and native nucleus pulposus (NP^native^). ^*∗*^*P* < 0.05, ^*∗∗*^*P* < 0.01, ^*∗∗∗*^*P* < 0.001, one-way ANOVA, Tukey's post hoc test.

Interestingly, the NP-induced changes in expression were almost completely blocked when NP was applied together with minocycline, the inhibitor of glial cells and macrophage activation (fold expression: IL1*β*; 5.19 ± 2.08, TNF; 1.55 ± 0.43, Csf1; 0.92 ± 0.15, CX3CL1; 2.01 ± 0.51, CX3CR1; 1.95 ± 0.45). No clear changes in expression were observed in the corresponding fat control experiments where the NP was applied onto the intrascapular adipose tissue (fold expression: IL1*β*; 3.68 ± 0.54, TNF; 1.99 ± 0.32, Csf1; 1.53 ± 0.25, CX3CL1; 2.51 ± 0.33, CX3CR1; 2.66 ± 0.47). Thus, the increase in expression, which was dependent on the contact between the NP tissue and the dorsal nerve roots, was attenuated by minocycline (Figures 3(a)–3(e)).

**Figure 4:**
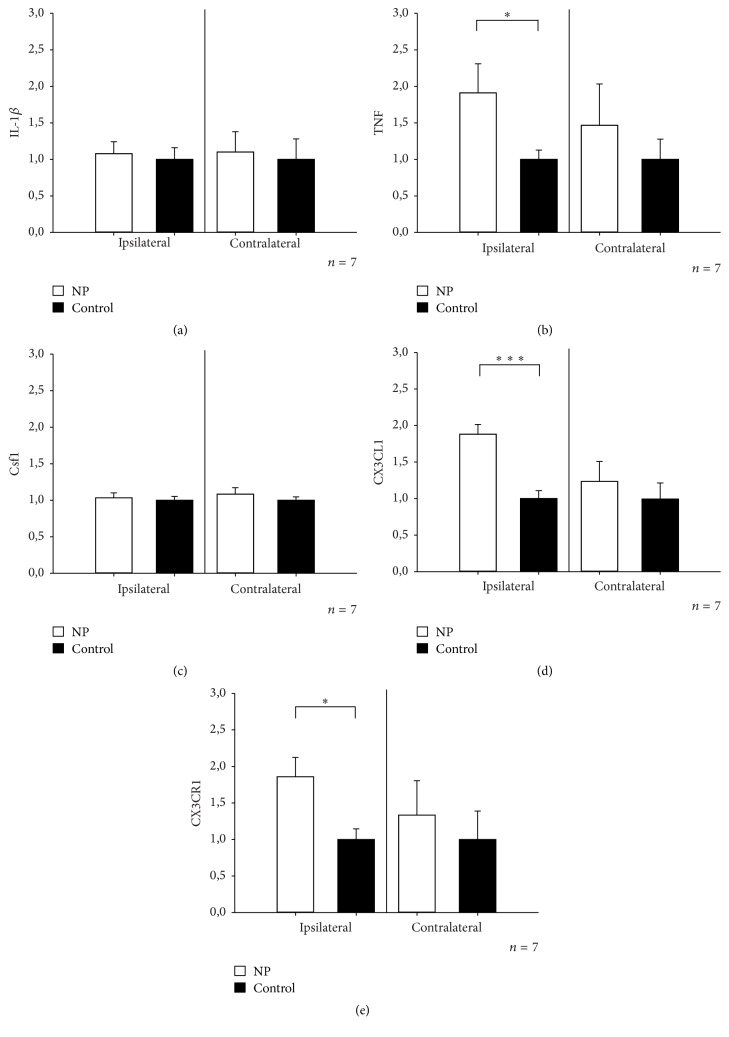
Fold expression of (a) IL-1*β*, (b) TNF, (c) Csf1, (d) CX3CL1, and (e) CX3CR1 in the ipsi- and contralateral DRG after NP application onto the dorsal nerve roots. ^*∗*^*P* < 0.05, ^*∗∗∗*^*P* < 0.001, Student's *t*-test.

After 3 hours, NP applied onto the left dorsal nerve roots induced an ipsilateral increase in the expressions of TNF, CX3CL1, and CX3CR1 in the DRGs (fold expression in ipsilateral DRGs: TNF; 1.91 ± 0.56, CX3CL1; 1.88 ± 0.13, CX3CR1; 1.86 ± 0.26; fold expression in contralateral DRGs: TNF; 1.47 ± 0.56, CX3CL1; 1.23 ± 0.27, CX3CR1; 1.33 ± 0.47) (Figures 4(a)–4(e)). Although NP was applied 5–15 mm proximal to the DRG, this seemed to have a clear effect on the DRG cells. The fold expressions of IL1-*β* and Csf1, however, were not increased (ipsilateral DRGs: IL-1*β*; 1.08 ± 0.16, Csf1; 1.03 ± 0.07; contralateral DRGs: IL-1*β*; 1.10 ± 0.28, Csf1; 1.08 ± 0.09).

## 4. Discussion

Our results showed that application of NP onto the dorsal nerve roots within minutes increased the C-fiber response in the dorsal horn. The changes in gene expression in the DRG suggest that the primary afferent nerve fibers may be affected by NP. In addition, proinflammatory cytokines released near the dorsal nerve roots could diffuse to the dorsal horn and affect postsynaptic cells and spinal microglia. Thus, the increase in excitability at the spinal level may be due to direct effects of NP on the primary nerve fibers, but also secondary changes induced by molecules from NP in postsynaptic WDR neurons. Previous findings indicate that the rapid effects of NP are induced by IL-1*β* and TNF action on dorsal horn lamina II ion channels and suppressed spinal GABA and glycine-induced currents [20, 21]. The effect of NP on nociceptive signaling has also been demonstrated in behavioral studies [22].

Previous observations show that application of NP onto the dorsal nerve roots may affect the expression of cytokines like IL-1*β* and TNF in the NP tissue [1]. In accordance with these earlier findings, the present data show a significant upregulation of IL-1*β* after application of NP onto the dorsal nerve roots in the NP tissue. However, in contrast to the observation of our earlier report, no clear upregulation of TNF was observed in the NP tissue this time. On the other hand, the present data showed a significant upregulation of TNF in the DRG.

Anyway, several lines of evidence suggest that IL-1*β* and TNF, but also Csf1, may be upregulated in NP or surrounding tissue following disc herniation [1, 3, 4, 23]. Therefore, it seems likely that persistent hyperexcitability in the pain pathways after disc herniation is associated with a local inflammatory process including activation of macrophages close to the nerve roots. Moreover, previous data show that IL-1*β* [24, 25], Csf1 [26–28], and CX3CL1 [22, 29, 30] can induce M1 microglia activation. For the first time, however, we showed that application of NP onto the dorsal nerve roots also increased the gene expression of CX3CL1 and CX3CR1 in the NP cells and in the ipsilateral DRGs. These observations suggest that local upregulation of IL-1*β*, Csf1, and CX3CL1 following disc herniation may activate microglia and induce pain [21]. Interestingly, no change in gene expression was observed in NP tissue following contact with intrascapular adipose tissue, suggesting a specific interaction between NP cells and neuronal tissue.

In accordance with the earlier observation that microglial inhibition may prevent hyperalgesia [16, 17, 31], minocycline attenuated the increase in nociceptive signaling induced by NP in the present study. Data from earlier in vitro studies also suggest that inhibition of MAPK p38 in human NP cells inhibits inflammation [14]. Hence, it is tempting to speculate that the antinociceptive effects of minocycline may be a result of reduced MAPK p38 phosphorylation in microglia.

However, the anti-inflammatory effect of minocycline may be due to other mechanisms as well, not only by inhibition of p38 phosphorylation. For instance, minocycline is reportedly an inhibitor of nitric oxide synthase [32] and 5-lipoxygenase [33] attenuates TNF expression [34] and suppresses NF-kappa B activity [35]. In addition, minocycline may directly affect Na^+^-channels in primary afferent neurons [36], which could explain the short lasting drop in C-fiber response following minocycline administration. Minocycline also attenuated the increase in the gene expression of IL-1*β*, Csf1, CX3CL1, and CX3CR1 in NP tissue. The alleviating effect of minocycline on nociceptive signaling following application of NP onto the dorsal nerve roots may therefore be related to this anti-inflammatory processes in NP.

The changes in gene expression were less pronounced in the DRG than in the NP tissue itself. Still, a significant increase in the expression of TNF, CX3CL1, and CX3CR1 was demonstrated, supporting the previous observation that local application of NP onto the primary afferents may change the gene expression in the DRGs [37]. The changes in gene expression were only observed on the ipsilateral side. This supports the hypothesis that the observed nociceptive effect is dependent on contact between NP tissue and the dorsal nerve roots.

Increased expression of CX3CL1 and its receptor CX3CR1 has previously been shown in the DRG after nerve injury [38]. Our observation that hyperexcitability in the pain pathways after application of NP may involve an upregulation of CX3CL1 signaling in the DRG supports these previous findings. Moreover, evidence exists that CX3CL1 mediates inflammatory pain through activation of DRG satellite glial cells [7]. However, recent discoveries challenge the previous notion that CX3CL1 is expressed in DRG neurons [39]. Thus, it seems likely that the observed CX3CL1 upregulation takes place in endothelial cells, possibly facilitating infiltration of monocytes or other leukocytes into the DRG [40].

The distance from the NP graft to the DRG in our animal model may be larger than the distance from the NP to the DRG in lumbar disc herniation in patients. Still, our data show that application of NP onto the dorsal nerve roots induces a significant upregulation of several genes in the ipsilateral DRG tissue, emphasizing the clinical relevance of our animal model. Moreover, previous data show that peripheral inflammation through circulating IL-1*β* may increase the expression, of, for example, Cox 2, in the spinal cord [41]. Notably, circulating mediators released in the peripheral tissue may therefore also influence central secondary WDR neurons.

## 5. Conclusion

In summary, the present data suggest that hyperexcitability in the pain pathways and the local inflammation after disc herniation may involve upregulation of CX3CL1 signaling in both the NP and the DRG. The molecular consequence of the upregulation of CX3CL1 remains to be investigated.

## Figures and Tables

**Table 1:** Primer sequences.

Primer	Sequence 5′ → 3′	Bp	% GC	Tm °C
IL1*β* forward	CGT GGA GCT TCC AGG ATG AG	20	60.0	59.4
IL1*β* reverse	CGT CAT CAT CCC ACG AGT CA	20	50.0	59.1
TNF forward	GCC ACC ACG CTC TTC TGT CTA	21	57.1	59.1
TNF reverse	TGA GAG GGA GCC CAT TTG G	19	57.9	59.6
Csf1 forward	GGG AAT GGA CAC CTA CAG ATT TTG	24	45.8	59.6
Csf1 reverse	AAA TTT ATA TTC GAT CAG GCA TGCA	25	32.0	59.7
CX3CL1 forward	TTG CAC AGC CCA GAT CAT TC	21	47.6	54.3
CX3CL1 reverse	CTG CGC TCT CAG ATG TAG GAA A	22	50.0	55.7
CX3CR1 forward	GTG GCC TTT GGG ACC ATC T	19	57.9	56.7
CX3CR1 reverse	CCA CCA GAC CGA ACG TGA A	19	57.9	56.6
*β*-actin forward	CTA AGG CCA ACC GTG AAA AGA	21	47.6	58.0
*β*-actin reverse	ACA ACA CAG CCT GGA TGG CTA	21	52.4	59.2
